# Executions by Electricity

**Published:** 1891-10

**Authors:** 


### HALL'S JOURNAL OF HEALTH

TRUTH DEMANDS NO SACRIFICE; ERROR CAN MAKE NONE.

Vol. 38. OCTOBER, 1891. No. 10.

### EXECUTIONS BY ELECTRICITY.

Without discussing the moral right of a State to deliberately take the life of an individual for any cause, let us briefly consider the new method of executing the death penalty in the State of New York, so recently resorted to and pronounced by the cortege of official spectators, both instantaneous and painless.

At first view, these desirable qualities would seem to answer the highest requirements of a killing machine, whether for man or beast. It is nevertheless, sad to contemplate that in this age, in most respects so far removed from the barbarisms of the past, the governing powers should adhere to the cruel and revengeful custom of taking the life of a human being for any offense, even the gravest, when as now, in all civilized communities, the means of effectually preventing his further evil doing, are at hand. Rightly estimated the want of such means furnishes the sole justification for enforcing the death penalty.

By a comparison of the electrical process with that of strangulation by hanging, the objection to the former over the latter is not limited to the mode of killing, although when it is considered that man is no less a spiritual than a temporal being—that he contains within himself —*i. e.*, the animal man—a spiritual selfhood—an ego which cannot die, the electrical process is objectionable in a sense which, from a merely physical standpoint, has met with little or no consideration.

There is something in the account which has been given to the public of the late triplicate executions in this State, that reminds one of the secret working of the Spanish Inquisition in its worst days. Here too, is the cruel enginery of torture and of death!—the "Dreadful note of preparation," the binding to the fateful chair! the shaved crown and deadly headpiece, and more terrible still, the solemn and mysterious chamber at whose threshold all hope is abandoned.

Then comes the dreadful shock that sends a fellow creature, all unprepared, to his last account. There is a fearful contortion of muscles, a spasm, and an effluvium of burning flesh that would delight the veriest cannibal, and the iron chair is ready for another victim in waiting.

These are refinements of cruelty worthy of the Satanic inventions of the dark ages. No humane citizen of the great and masterly State of New York, can contemplate them with composure. Away with the whole barbarous system of taking human life under the sanction of the law, say we. It is as inhuman in practice as it is unnecessary in fact. Let us as a people, turn from this offensive pageantry of crime and give ear to

"The still, sad music of HUMANITY."

"The greatest attribute of HEAVEN is MERCY; and 'tis the crown of justice and the glory, when it may kill with right, to save with pity."

